# Cholesterol Content, Fatty Acid Profile and Health Lipid Indices in the Egg Yolk of Eggs from Hens at the End of the Laying Cycle, Following Alpha-Ketoglutarate Supplementation

**DOI:** 10.3390/foods10030596

**Published:** 2021-03-11

**Authors:** Ewa Tomaszewska, Siemowit Muszyński, Anna Arczewska-Włosek, Piotr Domaradzki, Renata Pyz-Łukasik, Janine Donaldson, Sylwester Świątkiewicz

**Affiliations:** 1 Department of Animal Physiology, Faculty of Veterinary Medicine, University of Life Sciences in Lublin, Akademicka St. 13, 20-950 Lublin, Poland; 2 Department of Biophysics, Faculty of Environmental Biology, University of Life Sciences in Lublin, Akademicka St. 13, 20-950 Lublin, Poland; 3 Department of Animal Nutrition and Feed Science, National Research Institute of Animal Production, Krakowska St. 1, 32-083 Balice, Poland; anna.arczewska@izoo.krakow.pl (A.A.-W.); sylwester.swiatkiewicz@izoo.krakow.pl (S.Ś.); 4 Department of Commodity Science and Processing of Raw Animal Materials, Faculty of Animal Sciences and Bioeconomy, University of Life Sciences in Lublin, Akademicka St. 13, 20-950 Lublin, Poland; piotr.domaradzki@up.lublin.pl; 5 Department of Food Hygiene of Animal Origin, Faculty of Veterinary Medicine, University of Life Sciences in Lublin, Akademicka St. 12, 20-950 Lublin, Poland; renata.pyz@up.lublin.pl; 6 School of Physiology, Faculty of Health Sciences, University of the Witwatersrand, 7 York Road, Parktown, Johannesburg 2193, South Africa; janine.donaldson@wits.ac.za

**Keywords:** fatty acids, alpha-ketoglutarate, egg yolk, cholesterol

## Abstract

The current study aimed to assess the effects of dietary alpha-ketoglutarate (AKG) supplementation to laying hens on the fatty acid (FA) profile and cholesterol levels of the egg yolk at the end of production cycle. The experiment was performed on forty-eight Bovans Brown laying hens randomly assigned to either a control group (CONT) or a group supplemented with AKG. The CONT group was fed the basal diet, and the AKG group was fed the basal diet plus 1.0% AKG from the 31st until the 60th week of age, when FA profile, fat and cholesterol content of the egg yolks were determined. No significant changes in the cholesterol and total fat content of the egg yolks were observed. However, there were positive (the decrease in n-6 FA and the increase in MUFA), and negative (decrease in PUFA and n-3 FA, increase in TI and n-6/n-3 ratio) changes in FA profile following AKG supplementation. In conclusion, it was shown that dietary AKG after a 30-week long supplementation influence FA profile in egg yolk and its nutritional value.

## 1. Introduction

The increased consumption of highly processed food products rich in calories and fats (trans and saturated fatty acids, SFA) has resulted in an increase in the incidence of chronic diseases such as obesity, atherosclerosis, hypertension, cardiovascular diseases, type 2 diabetes and some forms of cancer [1]. A healthy diet is considered to have a moderate fat content and be rich in polyunsaturated fatty acids (PUFA), especially n-3 PUFA, at the expense of n-6 PUFA [1]. Eggs are a natural, unprocessed type of food with a high nutritional value and one of the highest nutrient concentration indices. However, eggs are highly variable in terms of quality, which is dependent on various internal and external features that in turn influence either the acceptance or rejection of this food by consumers [2,3]. The internal quality of the egg refers to the quality of the egg yolk and albumen, as well as their microbiological and functional properties. The nutritional value of the egg is primarily determined by its chemical composition and the content of exogenous ingredients. Albumen is a source of protein with high biological value, while yolk is rich in lipids. The dry weight of the yolk is about 50%, of which lipids constitute 32% of the dry matter. Lipids are an efficient source of metabolic energy and a rich source of substances of high biological and nutritional value, depending on the amount and type of fatty acids (FA) included in their composition. There are many different lipids within the egg yolk, including triglycerides, mono- and diglycerides, esters of cholesterol, cholesterol, phospholipids, and FA. In many recent years, the lipid composition of egg yolk was a primary consumer concern, due to the relationship between specific dietary lipids and the risk of development of coronary heart disease or cancer [4,5].

The lipid profile of the egg yolk can be affected by the genetics and age of the hens, as well as through modification of the composition of the feed provided to the hens [6,7,8]. The lipid composition of the egg yolk can be changed, in particular with regards to the FA profile, including the content of n-3 polyunsaturated fatty acids (PUFA) [4]. Numerous studies have been carried out on the influence of hen nutrition on the n-3 PUFA content of egg yolk. For this purpose, the addition of fish oils, fish meals, vegetable oils or oilseeds, mainly linseed and rapeseed, algae oil or some herbs are commonly included in the diet of hens [4,9,10,11]. Wu et al. [12] have shown that a diet enriched with n-3 PUFA changes the n-3 PUFA composition of the egg yolk. However, a proper balance in the consumption of n-3 and n-6 PUFA is expected. It has been proven that a ratio of n-6/n-3 of between 5:1 and 3:1 is optimal for human health [13]. The appropriate n-6/n-3 ratio plays a significant role in the amount of eggs consumed, along with the amount of cholesterol in the yolk [5].

Glutamine is considered a non-essential amino acid and makes up about 50% of the free amino acid pool in the bloodstream and plays an important physiological role in general metabolism, including that of lipids and fatty acids [14,15]. Glutamine contributes to muscle repair, prevents protein catabolism, improves nitrogen retention and functions as an immunomodulatory molecule [16,17,18]. The nutritive protective role of dietary glutamine has been highlighted in many previous experiments (in vitro, human and animal models, including birds and mammals) [14,19,20]. Alpha-ketoglutarate (AKG; 2-oxoglutaric acid, 2-Ox) is a glutamate and glutamine derivative, a precursor of arginine and other active compounds that are important in the regulation of protein metabolism in skeletal muscle [16]. AKG is a central molecule in the tricarboxylic acid cycle and is an essential co-substrate for a group of over 60 different, phylogenetically conserved enzymes, which catalyze hydroxylation reactions on various types of substrates, including proteins, nucleic acids, lipids, and metabolic intermediates. As AKG acts through many different mechanisms [16,21,22], it is widely used as a dietary additive in both human and veterinary medicine [16,23,24,25,26]. Reported specific health benefits include the improvement of the immune response, intestinal development or bone metabolism, which have been studied in a number of different species [27,28,29], but it also was effective in lowering blood cholesterol level and lipid metabolism regulation [26,30,31,32]. Fatty acids are created predominantly in the liver from acetyl coenzyme A (acetyl-CoA). AKG is intermediate in the pathway of the synthesis of FA from acetyl-CoA [33]. When AKG is added as a supplement, an increase is observed in the reaction between alanine and AKG, which generates glutamate and the puryvate, which are needed for the formation of acetyl-CoA, from which FA are synthetized. Synthetized FA are transported to the ovary by the bloodstream like other lipids and are deposited in egg yolk [34]. Furthermore, we have recently shown that AKG administered to laying hens decreased cholesterol content in blood and breast muscles of laying hens [35].

Still, there are no data on the effect of an AKG-enriched diet in layers on the FA profile or cholesterol content of the egg yolk. We hypothesized that dietary AKG, a derivative of glutamine and glutamate, would enhance protein synthesis, decrease cholesterol content, and serve as a substrate in fatty acid synthesis. We hypothesized that would also affect the fatty acid profile and cholesterol content of the egg yolk, thus influencing the nutritional value of the egg yolk. The objective of this study was to confirm the hypothesis presented above, where the effects of 30-weeks long dietary AKG supplementation to layer hens on the fatty acid profile of the egg yolk was evaluated in eggs in the late laying cycle.

## 2. Materials and Methods

### 2.1. Birds and Experimental Diets

The experiment was carried out using forty-eight 17-week-old Bovans Brown hens obtained from a commercial hatchery. Upon arrival, hens were housed in a poultry house in cages (two hens per cage) on a wire-mesh floor under controlled standard climate conditions. Each cage provided 3600 cm^2^ of total floor space and was equipped with a curtained nest area, a claw-shortening device, two perches, one nipple drinker and two feed troughs. During the pre-experimental period, lasting from the 18th to the 30th week of age, all hens— pre-laying (18–20 wks) and laying (21–30 wks)—were fed the same standard commercial diets; the laying diet was the same as that used during the experimental period (Supplementary Table S1). All diets met the nutritional requirements according to Polish recommendations [36]. The chemical composition of the feed materials was determined using conventional methods [37]. Calcium and phosphorus content were determined using flame atomic absorption spectrophotometry and the calorimetric method, respectively [31]. Rapeseed oil was used as the primary fat source in the experimental diets, the fatty acid profile of which is given in Supplementary Table S2 [7]. The experimental period ran from the 31st to the 60th week of age of the birds. At the start of the experimental period, the hens were randomly assigned to one of the two experimental groups: the control group (CONT group) or the group supplemented (in the diet) with AKG (AKG group), each comprising 12 replicate cages, with two birds per cage. The CONT group was fed the basal diet, and the AKG group was fed the basal diet plus 1.0% AKG (α-Ketoglutaric acid, #75890, Sigma-Aldrich, St. Louis, MO, USA). AKG (10 g/kg feed) was administered to the basal diet and was not considered when balancing the compound feed.

The supplemental AKG dose was chosen based on the literature, with the selection criterion being the most optimal performance in laying hens (feed intake, feed conversion per kilogram of eggs, and egg quality) and broiler chickens (humoral immune response, blood morphology) [17,18,20,38,39]. The glutamine concentration in the basal diet was not determined. During the experimental period, laying rate, egg weight, and feed intake were recorded. On the last two days of the study (at the end of 30th week of the experiment), two eggs from each cage were collected (n = 24 in each experimental group). Yolks were separated and frozen at −20 °C until determination of egg yolk fatty acid composition and cholesterol content.

### 2.2. Determination of Egg Yolk Fatty Acid Composition and Cholesterol Content

Analysis of the total fat content and fatty acid composition was performed according to the methods described in Domaradzki et al. [40]. Egg yolk samples were freeze-dried to a constant weight using an Alpha 1–4 LDplus freeze dryer (Martin Christ Gerfriertrocknungsanlagen, GmbH, Osterode am Harz, Germany). Total fat content (%) was gravimetrically determined using a Büchi B-811 extraction system (Büchi Labortechnik AG, Flawil, Switzerland), after Soxhlet extraction with n-hexane. Fatty acid methyl esters (FAMEs) were analyzed following fat extraction according to the methods described by Folch et al. [41] and prepared according to AOAC # Ce 2–66 method [37] using CG 3900 gas chromatograph (Varian, Walnut Creek, CA, USA) with FID detector. An automatic sampler with a flow rate of 2 mL/min was used to inject the sample (1 μL) onto a CP 7420 capillary column (Agilent Technologies, Santa Clara, CA, USA; 100 m in length, inner diameter 0.25 mm, film thickness 0.25 μm). Hydrogen was used as a carrier gas at a flow rate of 2 mL/min. The oven temperature was set from 50 °C (1 min) to 120 °C with a 30 °C/min rise, then to 160 °C (30 min) with a 2 °C/min rise, then to 200 °C (1 min) with a 1 °C /min rise, then to 250 °C (1 min) with a 5 °C/min rise. The temperature of the injector and the detector was 270 °C, the size of the injected samples was 1 µL and the split ratio 1:50. Identification and quantification of FAMEs was based on retention times corresponding to reference mixtures (Supelco Inc., Bellefonte, PA, USA; Larodan AB, Solna, Sweden). Each FAME (% of total FAMEs) was converted into the corresponding fatty acid (% of total fatty acids) using individual Sheppard factors (ShF). Partial sums, related indices and ratios of fatty acids were calculated according to the following formulas: long-chain n-6 fatty acids-LC n-6: sum of 20:2 n-6, 20:3 n-6, 20:4 n-6, 22:4 n-6 and 22:5 n-6; long-chain n-3 fatty acids-LC n-3: sum of 20:3 n-3, 20:4 n-3, 20:5 n-3, 22:5 n-3 and 22:6 n-3; long-chain polyunsaturated fatty acids-LC-PUFA: sum of LC n-6 and LC n-3; conjugated linoleic acid-ΣCLA: sum of 18:2 c9,t11, 18:2 t9,c11, 18:2 t7,c9, 18:2 t11,c13, 18:2 t12,c14, 18:2 t11,t13; ΣC18:2 trans: sum of non-conjugated 18:2 t,c/c,t/t,t isomers; ΣTFA: sum of monounsaturated fatty acids (MUFA) trans and ΣC18:2 trans [40]. Based on the proportions of particular fatty acids and their groups, the following health lipid indices were calculated: NV—nutritional value; S/P—saturation index, AI—atherogenicity index and TI—thrombogenic index [42]; h/H—hypocholesterolaemic/hypercholesterolaemic ratio [40,43].

Cholesterol content in the egg yolk (mg/g) was determined using gas chromatography with an internal standard (5-alpha-cholestan) according to the methods described by Hwang et al. [44]. Lipid extraction was performed according to the Folch method [41], and saponification was performed with 0.5 mL KOH in ethanol. Cholesterol was extracted with hexane. Sample solutions (5 µL) were analyzed using a gas chromatograph (GC-210, Shimadzu Corp., Kyoto, Japan) with operating conditions as follows: capillary column ZebronZB-5 (Phenomenex, Torrance, CA, USA; 30 m × 0.25 mm, 0.50 µm); flow rate of gas carrier (helium) 1.7 mL/min, column temperature 100 °C gradually increasing by 30 °C/min up to 150 °C and then by 15 °C/min up to 265 °C; injector and FID detector temperature 300 °C; split ratio 1:25, time of analysis 30 min [45].

All analyses were taken separately for each yolk sample the, and the average for each repetition cage was taken as the final result.

### 2.3. Statistical Analyses

Values are expressed as the mean ± SEM from n = 12 egg samples per each group. For statistical analyses, each individual cage was considered as an experimental unit. All variables were examined for normality (Shapiro–Wilk test) and homogeneity of variance (Levene test) and were analyzed using a two-tailed Student’s *t*-test, and a t-test with Welch’s correction when normally distributed data lacked equal variances or a non-parametric Mann–Whitney U test when there was a lack of normal distribution. For all tests, a *p*-value < 0.05 were established.

Principal component analysis (PCA) was also applied to the calculated values of the partial sum of fatty acids (Σ SFA, Σ MUFA, Σ PUFA, Σ n-3, Σ n-6) of all samples studied. The Kaiser–Meyer–Olkin measure (KMO) and the Bartlett’s test of sphericity were used to determine the significance of the PCA. The initial choice of principal components to explain the majority of the additive fatty acid profile variation of the data set was determined as those with eigen values greater than one, in accordance with Kaiser’s criterion, supported by Cattell’s scree plot analysis.

All calculations were carried out using Statistica software (v. 13.3, TIBCO Software Inc., Palo Alto, CA, USA).

## 3. Results

The diet had no effect on overall laying rate (95.8 ± 0.4% and 96.6 ± 0.4% in the Control and AKG group, respectively; *p* = 0.108) and daily feed intake (118 ± 1 g per hen and 117 ± 1 g per hen in the Control and AKG group, respectively; *p* = 0.226), as reported previously [35]. There were also no differences in egg mass (60.79 ± 0.98 g and 62.60 ± 0.59 g in the Control and AKG group, respectively; *p* = 0.120) and egg yolk mass (16.65 ± 0.32 g and 17.40 ± 0.37 g in the Control and AKG group, respectively; *p* = 0.134) between groups. The analysis of total fat and total cholesterol content showed no difference between groups (Table 1). Assessment of the egg yolk fatty acid composition included the determination of saturated fatty acids—SFA, odd-chain fatty acids—OCFA, monounsaturated fatty acids—MUFA cis, and polyunsaturated fatty acids—PUFA. Generally, AKG supplementation had no effect on total SFA, OCFA, increased total MUFA cis and trans, and decreased total PUFA n-6 and n-3 FA, influencing PUFA/SFA and n-6/n-3 ratios. The changes in determined health lipid indices were also observed. All changes in details are presented in Table 1.

**Figure 1:**
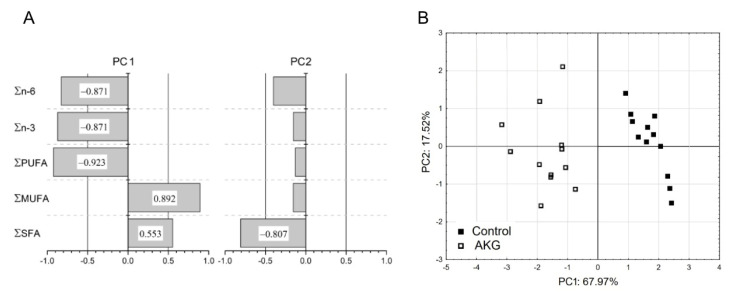
Results of the principal component analysis (PCA) for partial sums of fatty acids (Σ SFA, Σ MUFA, Σ PUFA, Σ n-3, Σ n-6) of the yolk of eggs obtained from hens in the control group and from hens supplemented with 1.0% AKG during a 30-week experimental period (31st–60th week of age), determined at the end of study. (**A**) Correlation between all the original variables included in the PCA of the first two principal components (PC). Correlations greater than 0.5 (absolute value) are considered significant. (**B**) PCA bi-plot (loading plot) for the PC1 and PC2 components.

In order to compare the partial sums of fatty acids in the yolk of eggs obtained from hens in the control and AKG groups and to analyze the variability in their analysis’s variables, a PCA was applied (Figure 1). The general Kaiser–Meyer–Olkin (KMO) measure of sampling adequacy was 0.817 (and between 0.707 and 0.891 for particular variables), and the Bartlett’s test of sphericity (Chi-squared value 66.553) reached statistical significance (*p*-value < 0.001), providing the basis for the application of the PCA analysis. The first two principal components (PC), showing eigenvalues greater than or close to 1 (3.40 and 0.88 for PCA 1 and PCA 2, respectively), explain 85.6% of the variation of the data. The first principal component PC1 (eigenvalue: 3.40) explained most of the variability of the data (68.00%) and was significantly negatively associated with Σ PUFA, Σ n-3 and Σ n-6 (correlation −0.923, −0.871, and −0.871, respectively) and positively corelated with Σ MUFA (correlation: 0.892) (Figure 1A). The second component PC2 (eigenvalue: 0.88) explained less of the variance (17.5%) and was only negatively correlated with Σ SFA (correlation: −0.807). As the eigenvalue of PC2 was slightly below 1 and therefore did not fully meet Kaiser’s criterion of inclusion, a Cattell’s scree plot (Supplementary Figure S1) was used to confirm that the usage of PC2 was appropriate. PCA loading plot for the first two components, which combined explain 85.6% of the variation of the data, showed that Control and AKG samples are totally separated by PC1 (Figure 1B). This was not observed for the second component PC2, which did not separate examined samples.

Table 2 shows the FAO (Food and Agriculture Organization of the United Nations) recommended levels of fat and FA as energy sources in the diet of an adult [46] and the corresponding EU recommendations for dietary intake of fat, FA, and cholesterol [47]. The most visible differences between eggs’ nutritional value can be observed for trans FA, however, their total intake of from yolk still will be marginal.

**Table 2:** Nutritional contribution of the egg yolk in terms of total fat, fatty acid content and cholesterol content to the adult diet, considering the consumption of mean-sized eggs from the control (mean egg yolk weight: 16.6 g) and AKG-supplemented (mean egg yolk weight: 17.4 g) hens.

Specification	Percent (%) of Energy Requirements Recommended by FAO ^1^	g/Day (for a 2000 Kcal Diet) ^2^	Mean Content (g) in Egg Yolk		Percent (%) of Contribution for a 2000 Kcal Diet	
			Control	AKG	Control	AKG
Total fat	20.0–35.0	44.0–78.0	4.77	4.95	6.1–10.8	6.3–11.3
Σ SFA	<10.0	<22.0	1.26	1.33	≥5.7	≥6.0
Σ MUFA	15.0–20.0	33.0–44.0	2.03	2.21	4.6–6.2	5.0–6.7
Σ PUFA	6.0–11.0	13.0–24.0	0.66	0.56	2.7–5.0	2.3–4.3
Σ n-6	2.5–9.0	5.6–20.0	0.56	0.49	2.8–9.9	2.4–8.7
Σ n-3	0.5–2.0	1.1–4.4	0.099	0.077	2.2–9.0	1.7–7.0
trans FA	<1.0	<2.2	0.003	0.006	≥0.15	≥0.27
EPA + DHA ^3^	250 mg	0.250	0.057	0.048	22.8	19.2
Cholesterol ^4^	<300 mg	<0.300	0.19	0.20	≥65.1	≥67.2

^1^ FAO [46]. ^2^ EU [47]. ^3^ The upper level of EPA (C20:5n-3 icosapentaenoic acid) and DHA (C22:6n-3, docosahexaenoic acid) consumption should not exceed 2 g/day. EPA was detected in examined samples in trace amounts). ^4^ WHO [48].

## 4. Discussion

The use of nutritional strategies to improve egg quality, specifically the FA profile, is well known and has been extensively studied [8,9]. It is difficult to compare the results obtained from the current study with data presented in the literature, since there are no available reports on AKG effects on egg yolk FA profile in laying hens and poultry in general. However, our study showed that AKG supplementation did not influence the total lipid and cholesterol content of the egg yolk, with amounts that correspond to those previously noted [49,50]. The FA profile of the egg yolk is composed of SFA and unsaturated FA, which include MUFA and PUFA (including n-3 and n-6). The basic SFA of the egg yolk include C14:0 (myristic acid), C16:0 (palmitic acid) and C18:0 (stearic acid). Whilst palmitic acid has been shown to have hypercholesterolemic effects, myristic acid and stearic acid are considered to be biologically inert. The PUFA in egg yolk, which are considered biologically to be the most important FA, include: C18:2n-6 (LA, linoleic acid), C18:3n-3 (ALA, linolenic acid), C20:5n-3 (EPA, timnodonic acid) and C22:6n-3 (DHA, cervonic acid). Linoleic acid is a substrate in the biosynthesis of other n-6 FA with longer chains and more double bonds, e.g., C20:4n-6 (ARA, arachidonic acid) and others [51]. The n-3 FA are among the most desirable FA because they have been shown to have a positive effect on inflammation and prevent chronic diseases, including heart disease and dementia. They are necessary for the proper functioning of the nervous system and cannot be synthesized by the body and must therefore be provided in the diet. The n-3 FA are especially important for the early development of central nervous system. The symptoms of n-3 FA deficiency in adults may include chronic fatigue, poor memory, mood disorders, circulatory disorders, and depression. Acids from the n-6 FA family are another group of PUFA, some of which (linoleic, arachidonic) are pro-inflammatory. They help to stimulate skin regeneration, maintain healthy bones, and ensure proper metabolism and proper condition of the reproductive system [52]. The n-6 FA have been shown to help in treating diabetic neuropathy and rheumatoid arthritis, as well as in reducing pain or morning stiffness.

The comparison of results from the current study with those from the literature is not only difficult, as previously mentioned, but also not really possible due to the lack of similar studies conducted on laying hens supplemented with AKG. The biological value of fat is primarily determined by the amount and type of FA it contains. We have identified a total of 23 types of FA, including 3 types of SFA, 2 types of OCFA, 8 types of MUFA, and 10 types of PUFA in the egg yolk. In the yolks assessed, irrespective of the AKG supplementation or lack thereof, MUFA predominated, followed by SFA and then PUFA. Long-chain PUFA (LC-PUFA) were also found. Taking individual groups of FA into account, trans FA were the minority. The percentage of SFA and OCFA were unchanged, irrespective of the AKG supplementation. Palmitic acid, C18:1n-9 (oleic acid) and C18:2n-6 (linoleic acid) were the most abundant FA noted in the egg yolk, irrespective of the AKG supplementation. This FA profile is in accordance with that presented in the literature [10,11].

The lack of AKG effect on palmitic acid, the most abundant SFA in the egg yolk, is beneficial from a nutritional point of view, since increased dietary and circulating concentrations of palmitic acid have been shown to increase the risk of cardiovascular disease [45]. Moreover, palmitic acid was detected at a lower level than that previously presented in the literature [10,11]. Higher dietary intake of OCFA (C15:0, pentadecylic acid, and C17:0, margaric acid), is associated with lower risks of cardiovascular diseases and liver diseases and lower mortality. Pentadecylic acid is primarily consumed through the diet, while margaric acid is readily produced de novo during liver-based α-oxidation and the elongation of pentadecylic acid [53]. The lack of alteration in OCFA in the egg yolks in our study, from the point of view of the risk of development of cardiovascular diseases, could be considered a good result.

As previously mentioned, MUFA cis were dominant in our egg yolks, the content of which increased following AKG supplementation. A diet rich in MUFA, including cis-vaccenic acid and oleic acid in particular, has been linked with decreased low-density lipoprotein (LDL) cholesterol and increased high-density lipoprotein (HDL) cholesterol, as well as a hypotensive effect, which are considered to be beneficial health effects [54]. C14:1c9 (myristoleic acid) has some anticarcinogenic effects [55], while C16:1c9 (palmitoleic acid) has been shown to have anti-inflammatory actions and improve insulin sensitivity in liver and skeletal muscles [56]. Taking into consideration the roles of the above-mentioned MUFA, an increase in their content in egg yolk could be considered beneficial for the health of consumers. Among the MUFA, AKG supplementation only decreased the C15:1 and C17:1c9 (palmitoleic acid), which are present in a very minor amount. Cis-MUFA (C14–C22) have been shown to inhibit the enzyme topoisomerase I, a key enzyme in the breakdown and repair of DNA strands. The enzyme is necessary for topological changes in DNA for key cellular processes (replication, transcription, recombination) [57]. It seems that the decrease in C15:1 observed in the AKG group, a known bacterial FA, cannot be considered beneficial.

The presence of C17:1c9 (heptadecenoic acid) in the egg yolk, irrespective of AKG supplementation in our study, may be a result of the microbial activity in the intestinal tract of the hens, which was additionally modified by AKG [35]. Existing data regarding the FA profile of egg yolk indicate that further studies involving the intestinal microbiome are needed with regards to the role of the intestinal microbiome on yolk lipid composition.

PUFA were abundant in the egg yolks assessed in our study, the percentage of which significantly decreased following AKG supplementation, due to a decrease in almost all of the PUFA detected, except C20:3n-6 (DGLA, dihomo-γ-linolenic) and C22:5n-6 (DPA n-6, osbond acid). DGLA is formed in the body of mammals from C18:3n-6 (GLA, γ-linolenic acid), while DPA n-6 is formed through the stepwise elongation and desaturation of C20:4n-6 (AA, arachidonic acid). Both precursors were decreased in our study. Since eggs are a primary dietary source of these PUFA, the fact that DGLA and DPA n-6 acid were not decreased in our study can be considered as beneficial. DGLA gives rise to eicosanoids that have anti-inflammatory, anti-cancer, vaso-relaxing and anti-aggregation properties [58]. Our study also showed that all the main n-6 PUFA were decreased in the egg yolk after AKG supplementation to laying hens. C18:2n-6 (LA, linoleic acid) is an essential n-6 PUFA, which is needed for normal growth and development, although it is non-functional in brain development due to its low concentration [59]. LA is also a precursor of oxidized LA metabolites, which are lipid mediators that regulate pain and inflammatory signals in peripheral tissue [60]. GLA is pro-inflammatory, but it can also act as an anti-inflammatory factor. DGLA, formed from GLA, is a precursor of prostaglandins and thromboxane, both of which play an anti-inflammatory role. DGLA also has an antithrombotic effect [61]. Among detected n-3 PUFA, the highest decrease was observed in C18:3n-3 (ALA, α-linolenic acid), C22:5 n-3 (DPA n-3, clupanodonic acid) and C22:6n-3 (DHA). ALA is an essential PUFA, from which other omega-3 FA can be formed. Clupanodonic acid plays a role in the inflammatory response [62], while DHA is a major PUFA in the phospholipids of the brain and the retina [63]. There is evidence that GLA, in combination with DHA and C20:5n-3 (EPA) can reduce high blood pressure [64].

The decrease in n-3 and n-6 PUFA, in particular, in the egg yolk following AKG supplementation to hens in the current study obviously resulted in a decrease in the sum of n-3 and n-6 PUFA, while the ratio of n-6/n-3 PUFA increased. Both n-6 and n-3 PUFA compete for the same enzymes but have different biological roles in humans; hence, a correct balance between them is of considerable importance. n-3 FA are among the most desirable FA, because they have been shown to have a positive effect on inflammation and the prevention of chronic diseases, including heart disease and dementia. The relationship between n-3 PUFA consumption and the lowering of the risk of coronary heart disease is complicated, since exceeding a certain amount of n-3 PUFA intake would not improve the risk [65]. On the other hand, excessive consumption of PUFA increases the concentration of very reactive free radicals formed during FA oxidation. In situations where there is an insufficient concentration of antioxidants, the increased level of free radicals increases the risk of structural damage to DNA and carcinogenesis [66]. DHA and AA belong to the group of LC-PUFA. The sum of LC-PUFA, as well as the sum of the individual families of LC-PUFA (n-3 and n-6), were decreased in the egg yolk in the current study.

Ratios of PUFA/SFA and n-6/n-3 are commonly used to assess the nutritional value of fat. Dietary ratios of PUFA to SFA above 0.45 and n-6 to n-3 of below 4 are considered appropriate in order to protect against the development of ischemic heart disease [67].

The n-6/n-3 ratio in the egg yolks from the hens in the current study was slightly higher than 4, irrespective of the AKG supplementation. The PUFA/SFA ratio, however, was above 0.45 in the egg yolks from the control group and slightly lower than 0.45 in the AKG group. The content of palmitic acid, the most abundant SFA, was detected at a lower level in the egg yolks from our hens compared to that stated in the literature [10], while the sum of OCFA observed in our study was higher [10]. It is evident that there are numerous conflicting results with regards to the FA profile of egg yolk.

PCA confirmed that AKG supplementation influenced the content of FA in the egg yolk. The loading plot presented in Figure 1B reports that PC1 clearly separated and clustered Control and AKG samples. PC1 can be considered as “pro-health” component of FA (strong absolute correlation with Σ MUFA, Σ PUFA, Σ n-3 and Σ n-3, all of which were significantly affected by AKG treatment). The second component PC2, which can be considered as the “anti-health” component of FA (strong and only significant correlation with Σ SFA), did not separate examined samples, demonstrating that yolk Σ SFA content shows a low ability of differentiating eggs laid by AKG-supplemented hens, confirming that the main effect of AKG revealed itself in lipid profile of MUFA and PUFA.

AKG supplementation to our hens was shown to increase the TI and S/P indices and decrease the h/H index, which in turn relates to a deterioration in the nutritional quality of the egg yolks [43]. These results were not fully expected since fat with lower TI and AI indices and a higher h/H index has better nutritional quality.

Yolk cholesterol content has become an important issue to consumers, since cholesterol is synthesized by the human body and consumers have been advised to avoid the consumption of dietary cholesterol in order to prevent chronic diseases, including coronary heart disease. More recently, it has been determined that exogenous cholesterol actually represents a very small amount of hematic cholesterol [68]. Nevertheless, the recommended cholesterol intake should not exceed 300 mg per day for adults [5,48]. Moreover, if fat intake is controlled, there is no need to severely restrict the intake of egg yolks, as long as the intake of SFA, which are a significant source of dietary cholesterol, is controlled [5,47].

Although the present study has some limitations—such as using one dose of AKG, which was selected based on hens welfare and therefore cannot be categorically considered as the most favorable in terms of its effect of FA profile, as well as using a single time point in laying cycle in which eggs were analyzed—in our opinion, the obtained results clearly showed that AKG, glutamine precursor, can influence FA metabolism in layers and FA profile in egg yolk. While it cannot be stated that AKG supplementation exerted unequivocally positive effects, this study showed that FA profile of yolk egg can be modified not only by the change of primary dietary fat source. Although general mechanism of AKG in metabolism is known, it must be highlighted that changes in the fatty acids profile can result not only from the AKG supplementation, but also from the mutual relationships of their metabolic pathways. For this reason, further studies involving other AKG doses and laying periods are needed.

## 5. Conclusions

In conclusion, AKG supplementation affected the FA composition of the egg yolk, but it is difficult to clearly indicate if the changes are in fact beneficial from a consumer’s point of view. On one hand, there were positive effects, like the decrease in n-6 FA and the increase in MUFA, of which egg yolk is a valuable source. However, on the other hand, there were also negative outcomes (decrease in PUFA and n-3 FA, increase in TI and n-6/n-3 ratio) following AKG supplementation. Taking into consideration all of the abovementioned results, it is clear that further studies are needed, including detailed analyses of other indices of egg quality.

## Figures and Tables

**Table 1:** The effect of dietary AKG supplementation to laying hens during a 30-week experimental period (31st–60th week of age) on egg yolk total fat, cholesterol content (%), yolk fatty acid composition (% of total fatty acids) and health lipid indices determined at the end of study.

Item	Control	AKG	*p*-Value
Total fat content, %	28.64 ± 0.13	28.46 ± 0.06	0.238
Total cholesterol, mg/g of yolk	11.73 ± 0.17	11.59 ± 0.11	0.471
Fatty acids			
C14:0	0.36 ± 0.02	0.37 ± 0.01	0.977
C16:0	24.55 ± 0.14	24.99 ± 0.23	0.126
C18:0	6.92 ± 0.06	7.01 ± 0.08	0.751
Σ SFA	31.89 ± 0.17	32.37 ± 0.19	0.073
C15:0	0.06 ± 0.01	0.06 ± 0.01	0.757
C17:0	0.18 ± 0.01	0.19 ± 0.02	0.501
Σ OCFA	0.24 ± 0.01	0.25 ± 0.02	0.627
C14:1c9	0.07 ± 0.01	0.08 ± 0.01	0.021
C15:1	0.09 ± 0.01	0.08 ± 0.01	0.016
C16:1c7	0.91 ± 0.03	0.97 ± 0.03	0.207
C16:1c9	2.98 ± 0.08	3.39 ± 0.08	0.001
C17:1c9	0.21 ± 0.01	0.18 ± 0.01	0.042
C18:1c9	44.49 ± 0.33	46.16 ± 0.27	<0.001
C18:1c11	2.24 ± 0.04	2.44 ± 0.03	<0.001
C20:1c11	0.22 ± 0.01	0.20 ± 0.01	0.029
Σ MUFA cis	51.23 ± 0.26	53.50 ± 0.29	<0.001
C18:2n-6	11.67 ± 0.26	9.69 ± 0.18	<0.001
C18:3n-6	0.09 ± 0.01	0.07 ± 0.01	0.008
C20:2n-6	0.08 ± 0.01	0.07 ± 0.01	0.047
C20:3n-6	0.13 ± 0.01	0.11 ± 0.01	0.069
C20:4n-6	1.73 ± 0.03	1.56 ± 0.01	<0.001
C22:4n-6	0.14 ± 0.01	0.11 ± 0.01	<0.001
C22:5n-6	0.23 ± 0.02	0.23 ± 0.01	0.751
Σ PUFA n-6	14.04 ± 0.27	11.84 ± 0.15	<0.001
C18:3n-3	0.91 ± 0.03	0.59 ± 0.01	<0.001
C22:5n-3	0.15 ± 0.01	0.12 ± 0.01	0.002
C22:6n-3	1.44 ± 0.03	1.17 ± 0.01	<0.001
Σ PUFA n-3	2.50 ± 0.06	1.87 ± 0.02	<0.001
Σ PUFA	16.55 ± 0.27	13.71 ± 0.23	<0.001
Σ LC-PUFA	3.91 ± 0.07	3.36 ± 0.02	<0.001
Σ LC n-6	2.31 ± 0.05	2.08 ± 0.02	<0.001
Σ LC n-3	1.59 ± 0.03	1.29 ± 0.01	<0.001
Σ MUFA trans	0.08 ± 0.01	0.15 ± 0.01	<0.001
PUFA n-6/n-3	5.65 ± 0.12	6.32 ± 0.06	<0.001
PUFA/SFA	0.52 ± 0.01	0.42 ± 0.01	<0.001
S/P	0.47 ± 0.01	0.48 ± 0.01	0.022
TI	0.79 ± 0.01	0.84 ± 0.01	<0.001
AI	0.38 ± 0.01	0.39 ± 0.01	0.062
h/H	2.44 ± 0.03	2.35 ± 0.03	0.036
NV	0.44 ± 0.01	0.45 ± 0.01	0.156

Values are expressed as the mean ± SEM from n = 12 samples per each group. Only FA with mean content ≥ 0.05% are specified. Presented Σ of particular group of FA also included FA < 0.05%. SFA—saturated fatty acids, OCFA—odd-chain fatty acids, MUFA—monosaturated fatty acids, PUFA—polyunsaturated fatty acids, LC-PUFA—long chain polyunsaturated fatty acids, S/P—saturation index, TI—thrombogenic index, AI—atherogenicity index, h/H—hypocholesterolaemic/hypercholesterolaemic ratio, NV—nutritional value.

## Data Availability

The data presented in this study are available on request from the corresponding author.

## Supplementary Materials

The following are available online at https://www.mdpi.com/2304-8158/10/3/596/s1: Table S1: Composition and nutrient content of experimental diet, g/kg dry matter; Table S2: Fatty acid profile of rapeseed oil, used as the primary fat source in the experimental diets, % of total fatty acids; Table S3: Physiological name, chemical name and common name of all fatty acids assessed in egg yolk; Figure S1: Scree plot showing eigenvalues for each principal component PC after performing PCA.
