# Supplementary material for: Cholesterol Content, Fatty Acid Profile and Health Lipid Indices in the Egg Yolk of Eggs from Hens at the End of the Laying Cycle, Following Alpha-Ketoglutarate Supplementation
Source: Foods. 2021 Mar 11;10(3):596. doi: 10.3390/foods10030596 (PMC8001726; doi:10.3390/foods10030596)
Supplement: Supplementary file 1 [file foods-10-00596-s001.pdf]

Table S1. Composition and nutrient content of experimental diet, g/kg dry matter.

Ingredients	Content
Ingredient (g/kg)	
Corn	422.10
Wheat	210.00
Soybean meal	236.00
Rapeseed oil	20.00
Limestone	90.00
Monocalcium phosphate	12.50
NaCl	3.00
DL-Methionine	1.40
Calcium phosphate	0.60
Vitamin-mineral premix[1]	5.00
Nutrients composition[2]	
Metabolizable energy, MJ/kg	11.60
Crude protein	170.00
Lys	8.35
Met	4.10
Cys	2.62
Ca	37.00
Total P	6.15
Available P	3.90

[1] The premix provided per 1 kg of diet: vitamin A, 10,000; vitamin D3, 3,000 IU; vitamin E, 50 IU; vitamin K3, 2 mg; vitamin B1, 1; vitamin B2, 4 mg; vitamin B6, 1.5; vitamin B12, 0.01 mg; Ca-pantotenate, 8 mg; niacin, 25 mg; folic acid, 0.5 mg; choline chloride, 250 mg; manganese, 100 mg; zinc, 50 mg; iron, 50 mg; copper, 8 mg; iodine, 0.8 mg; selenium, 0.2 mg, cobalt, 0.2 mg.

[2] Metabolizable energy (ME) calculated as a sum of the ME content of components.

Table S2. Fatty acid profile of rapeseed oil, used as the primary fat source in the experimental diets, % of total fatty acids.

Item	Content
C 8:0	0.0
C 10:0	0.0
C 12:0	0.0
C 14:0	0.0
C 16:0	4.75
C 16:1	0.20
C 18:0	1.44
C 18:1	59.5
C 18:2 n-6	19.6
C 18:3 n-3	13.3
C 20:4 n-6	0.0
C 20:5 (EPA)	0.04
C 22:6 (DHA)	0.0
Σ SFA	6.95
Σ UFA	93.0
Σ MUFA	60.0
Σ PUFA	33.1
Σ PUFA n-6	19.7
Σ PUFA n-3	13.4
PUFA n-6/n-3	1.47

EPA – eicosapentaenoic acid, DHA – docosahexaenoic acid, SFA – saturated fatty acids, UFA – unsaturated fatty acids, MUFA – monosaturated fatty acids, PUFA – polyunsaturated fatty acids.

Table S2. Physiological name, chemical name and common name of all fatty acids assessed in egg yolk.

Physiological name	Chemical name	Common name
C14:0	tetradecanoic acid	myristic acid
C16:0	hexadecanoic acid	palmitic acid
C18:0	octadecanoic acid	stearic acid
C15:0	pentadecanoic acid	pentadecylic acid
C17:0	heptadecanoic acid	margaric acid
C14:1c9	tetradecenoic acid	myristoleic acid
C15:1	cis-10-pentadecenoic acid	ginkgolic acid
C16:1c7	cis-7-hexadecenoic acid	palmitoleic acid
C16:1c9	cis-9-hexadecenoic acid	palmitoleic acid
C17:1c9	cis-9-heptadecenoic acid	heptadecenoic acid
C18:1c9	cis-9-octadecenoic acid	oleic acid
C18:1c11	cis-11-octadecenoic acid	vaccenic acid
C20:1c11	cis-11-eicosanic acid	gondoic acid
C18:2n-6	octadecadienoic acid	LA, linoleic acid
C18:3n-6	octadecatrienoic acid	GLA, γ-linolenic acid
C20:2n-6	cis-11-eicodienoic acid	eicosadienoic acid
C20:3n-6	cis-11-eicosatrienoic acid	DGLA, dihomo-γ-linolenic acid
C20:4n-6	eicosatetraenoic acid	AA, arachidonic acid
C22:4n-6	docosatetraenic acid	AdA, adrenic acid
C22:5n-6	docosapentaenoic acid	DPA n-6, osbond acid
C18:3n-3	octadecatrienoic acid	ALA, α-linolenic acid
C22:5n-3	docosapentaenoic acid	DPA n-3, clupanodonic acid
C22:6n-3	docosahexaenoic acid	DHA, cervonic acid

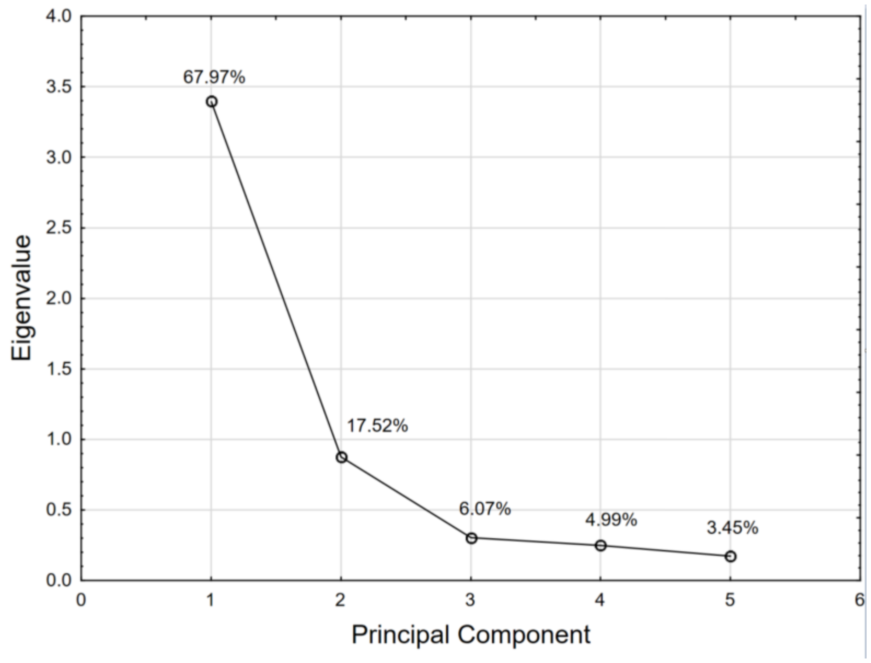

Figure S1. Scree plot showing eigenvalues for each principal component PC after performing PCA. Values on plot shows the precentage of total varience explained by each PC.
